# SARS-CoV-2 fusion-inhibitory lipopeptides maintain high potency against divergent variants of concern including Omicron

**DOI:** 10.1080/22221751.2022.2098060

**Published:** 2022-07-21

**Authors:** Yuanmei Zhu, Xiaojing Dong, Nian Liu, Tong Wu, Huihui Chong, Xiaobo Lei, Lili Ren, Jianwei Wang, Yuxian He

**Affiliations:** NHC Key Laboratory of Systems Biology of Pathogens, Institute of Pathogen Biology and Center for AIDS Research, Chinese Academy of Medical Sciences and Peking Union Medical College, Beijing, People’s Republic of China

**Keywords:** SARS-CoV-2, variants of concern, Omicron, fusion inhibitor, lipopeptide

## Abstract

The emergence of SARS-CoV-2 Omicron and other variants of concern (VOCs) has brought huge challenges to control the COVID-19 pandemic, calling for urgent development of effective vaccines and therapeutic drugs. In this study, we focused on characterizing the impacts of divergent VOCs on the antiviral activity of lipopeptide-based fusion inhibitors that we previously developed. First, we found that pseudoviruses bearing the S proteins of five VOCs (Alpha, Beta, Gamma, Delta, and Omicron) and one variant of interest (Lambda) exhibited greatly decreased infectivity relative to the wild-type (WT) strain or single D614G mutant, especially the Omicron pseudovirus. Differently, the most of variants exhibited an S protein with significantly enhanced cell fusion activity, whereas the S protein of Omicron still mediated decreased cell–cell fusion. Next, we verified that two lipopeptide-based fusion inhibitors, IPB02V3 and IPB24, maintained the highly potent activities in inhibiting various S proteins-driven cell fusion and pseudovirus infection. Surprisingly, both IPB02V3 and IPB24 lipopeptides displayed greatly increased potencies against the infection of authentic Omicron strain relative to the WT virus. The results suggest that Omicron variant evolves with a reduced cell fusion capacity and is more sensitive to the inhibition of fusion-inhibitory lipopeptides; thus, IPB02V3 and IPB24 can be further developed as potent, broad-spectrum antivirals for combating Omicron and the potential future outbreak of other emerging variants.

## Introduction

The continuing worldwide spread of SARS-CoV-2 has resulted in the emergence of many variants of concern (VOC) and interest (VOI). These variants affect virus’s properties, including transmission ability, disease severity, vaccine performance, as well as the therapeutic medicines and diagnostic tools [1–3]. The previous four VOCs are Alpha (B.1.1.7), Beta (B.1.351), Gamma (P.1), and Delta (B.1.617.2), which can lead to a new wave of pandemic and thousands of deaths. In November 2021, a highly transmissible VOC, designated Omicron (B.1.1.529), emerged and caused a great disturbance all over the world [4,5]. The Omicron strain can spread several times faster than any previous variants, and thus it quickly overtakes Delta to become the predominant variant worldwide. Importantly, it has been recognized that Omicron can seriously impair the clinical efficacy of many vaccines and antibody therapies and cause a large number of breakthrough infection or re-infection [5–7]. Therefore, it is urgent to develop effective and broad-spectrum antivirals for combating the Omicron epidemic.

**Figure 1.**
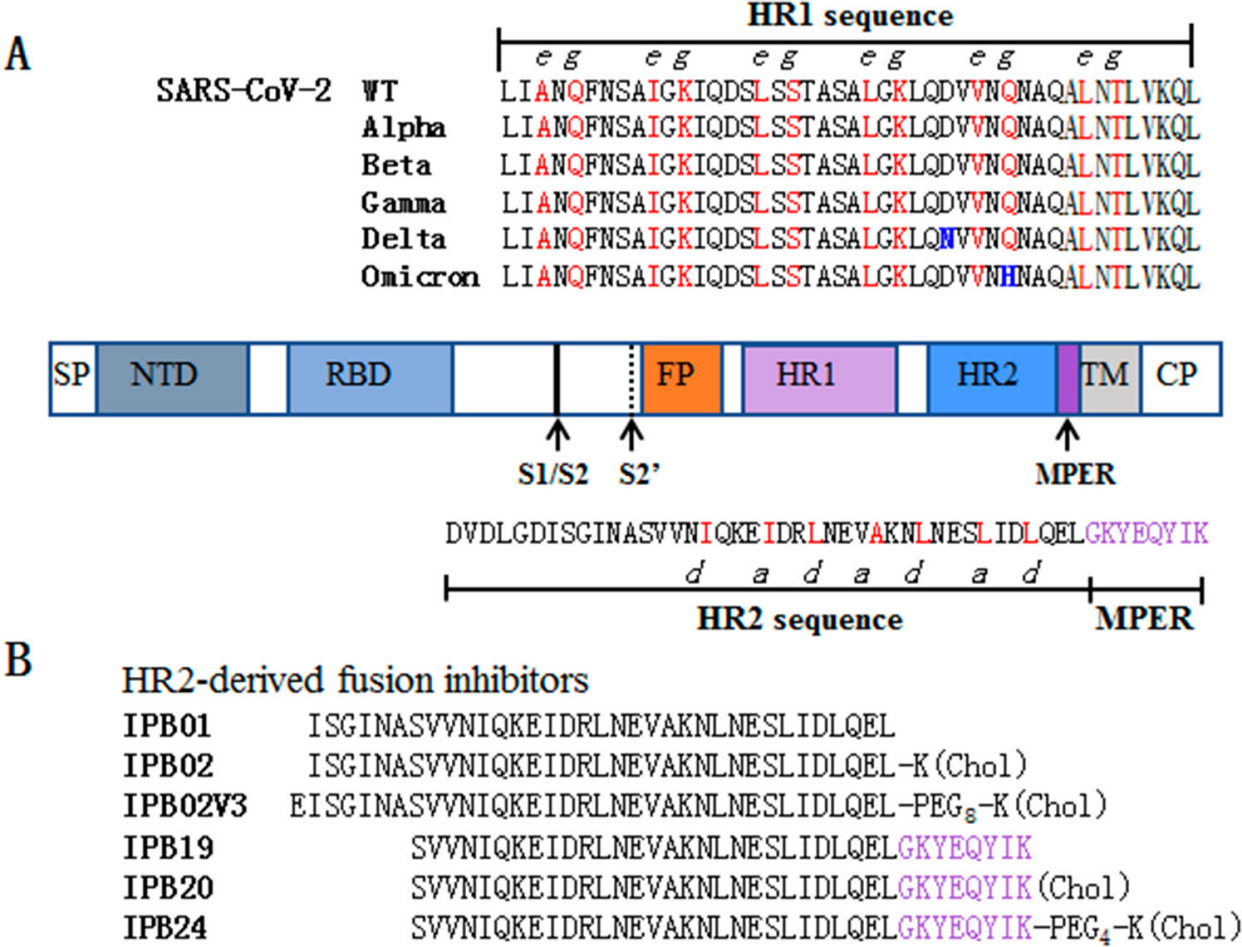
Schematic diagram of SARS-CoV-2 S protein and fusion inhibitors. (A) Functional domains of the S protein and HR1/HR2 sequences. SP, signal peptide; NTD, N-terminal domain; RBD, receptor-binding domain; FP, fusion peptide; HR1, heptad repeat 1 region; HR2, heptad repeat 2 region; MPER, membrane-proximal external region; TM, transmembrane domain; CP, cytoplasmic peptide. The S1/S2 and S2’ cleavage sites and MPER are marked with arrow. The HR1 and HR2 core sequences as well as the MPER sequence are listed, in which the potential residues that mediate the HR1–HR2 interactions in 6-HB are coloured in red. (B) The HR2-MPER sequence-derived fusion inhibitor peptides and lipopeptides. The MPER amino acids are coloured in purple. Chol, cholesterol; PEG8 or PEG4, 8-unit or 4-unit polyethylene glycol.

Like many other coronaviruses (CoVs), SARS-CoV-2 employs its trimeric spike (S) protein for cell entry through either a cytoplasmic or endosomal membrane fusion pathway. As a typical class I fusion protein, S protein is composed of an N-terminal S1 subunit and a C-terminal S2 subunit, which respectively mediate the receptor binding and membrane fusion [8,9]. It is known that the formation of a six-helical bundle (6-HB) structure between the heptad repeat 1 (HR1) and heptad repeat 2 (HR2) domains of S2 juxtaposes the viral and cell membranes for fusion reaction, and blocking the folding of the viral 6-HB core is an effective strategy for the development of antivirals as fusion inhibitors [10,11]. In the outbreak of COVID-19, we quickly developed a group of HR2 sequence-based SARS-CoV-2 fusion inhibitor lipopeptides, including IPB02V3 and IPB24 illustrated in Figure 1, which possess highly potent antiviral activity [11–14]. Considering the current Omicron epidemic and the potential of emergence of additional VOCs, as well as that we are in the process to develop a lipopeptide inhibitor for clinical use, it is fundamentally important to know the impact of divergent VOCs on the inhibitory sensitivity of the fusion inhibitors. In this study, we first analysed the functionalities of S proteins of divergent variants to mediate infection and membrane fusion; then, the antiviral activities of IPB02V3 and IPB24 against Omicron and other variants were determined. The data demonstrate that the Omicron variant has significantly decreased cell fusion capacity and is highly sensitive to the inhibitions of two fusion inhibitors.

## Materials and methods

### Plasmids, cells and inhibitors

Plasmids encoding the mutant spike protein of SARS-CoV-2 (Alpha, Beta, Gamma, Delta, Lambda, and Omicron) were kindly provided by Linqi Zhang at the Tsinghua University (Beijing, China). Plasmids encoding DSP_1–7_ and DSP_8–11_ were a kind gift from Zene Matsuda at the Institute of Medical Science of the University of Tokyo (Tokyo, Japan). 293T, Huh-7, and Vero cells were purchased from the American type culture collection (ATCC) (Rockville, MD, USA); 293T/ACE2 cells that stably express human ACE2 were generated and preserved in our laboratory. Cells were cultured in complete growth medium consisting of Dulbecco’s minimal essential medium (DMEM) supplemented with 10% fetal bovine serum (FBS), 100 U/ml of penicillin–streptomycin, 2 mM L-glutamine, and 1 mM sodium pyruvate under 37°C and 5% CO2. Two SARS-CoV-2 fusion-inhibitory lipopeptides, IPB02V3 and IPB24, were synthesized and used in our previous studies [12,13].

### Single-cycle infection assay

The infectivity of divergent SARS-CoV-2 pseudoviruses in 293T/ACE2 or Huh-7 cells was determined by a single-cycle infection assay as described previously [13]. Briefly, SARS-CoV-2 pseudoviruses were generated by cotransfecting 293T cells with a wild-type (WT) or mutant S protein-expressing plasmid and a backbone plasmid (pNL4-3.luc.RE) that encodes an Env-defective, luciferase reporter-expressing HIV-1 genome. After 48 h culture, cell supernatants containing released virions were collected and stored at −80°C. To measure the inhibitory activities of IPB02V3 and IPB24, a serially 3-fold diluted lipopeptide was incubated with an equal volume of pseudoviruses at 37°C for 30 min, and the lipopeptide-pseudovirus mixture was then added to 293T/ACE2 or Huh-7 cells (10^4^ cells/well). After incubation at 37°C for 48 h, the cells were harvested and lysed in reporter lysis buffer, followed by the measurement of luciferase activity using luciferase assay reagents and a luminescence counter (Promega, Madison, WI, USA).

### Cell–cell fusion assay

SARS-CoV-2 S protein-mediated cell–cell fusion activity was determined by a dual-split-protein (DSP)-based cell–cell fusion assay as described previously [11]. In brief, a total of 1.5 × 10^4^ 293T cells (effector cells) were seeded in a 96-well plate and 1.5×10^5^/ml 293T/ACE2 cells (target cells) were seeded in a 10-cm culture dish, and then the cells were incubated at 37°C overnight. The effector cells were cotransfected with a mixture of a SARS-CoV-2 S-expressing plasmid and a DSP_1–7_ plasmid, the target cells were transfected with a DSP_8–11_ plasmid, and then the cells were incubated at 37°C for 24 h. To measure the inhibitory activities of IPB02V3 and IPB24, a serially 3-fold-diluted lipopeptide was added to the effector cells and incubated for 1 h, while the target cells were resuspended at 3 × 10^5^/ml in prewarmed culture medium containing EnduRen live cell substrate (Promega) at a final concentration of 17 ng/ml and incubated for 30 min. Then, 3 × 10^4^ of target cells were transferred to the effector cells and the cell mixture was spun down to facilitate cell–cell contact. Luciferase activity was measured at different time points as described above.

### Inhibition of live SARS-CoV-2 infection

The inhibitory activities of IPB02V3 and IPB24 against authentic SARS-CoV-2 (WT or Omicron variant) infection were determined as described previously [13]. In brief, a serially 3-fold diluted lipopeptide was incubated with 0.1 MOI of live WT or Omicron virus at 37°C for 1 h, and the lipopeptide-virus mixture was then added into Vero cells that were seeded in a 96-well plate one day before infection at a concentration of 1×10^4^ cells/well. After 1 h incubation, cell supernatants containing the lipopeptide and virus were replaced with opti-MEM containing 1% bovine serum albumin (BSA) and cultured at 37°C for 24 h. Next, the cell supernatants were collected and viral RNA was extracted with a Direct-zol RNA MiniPrep kit (Zymo Research, CA, USA). Viral copy numbers (VCN) were measured by RT–PCR using primers and probe targeting the specific N gene of SARS-CoV-2. PCR amplification conditions were 50°C, 15 min, 95°C, 3 min; 95°C, 15 s, 60°C, 45 s+ Plate Read, 50 cycles. The copies of the virus were calculated according to the standard curve, and percent inhibition was obtained by dividing the number of copies of the virus in the vehicle control group.

### Statistical analysis

The percent inhibition of virus infection and 50% inhibitory concentration (IC_50_) of inhibitors were calculated using GraphPad Prism 6 software (GraphPad Software Inc., San Diego, CA, USA). Statistical comparisons of pseudovirus infections and S protein-mediated cell–cell fusion activities were conducted by one-way ANOVA with Dunnett's multiple comparisons test (*, *P* < 0.05; **, *P* < 0.01; ***, *P* < 0.001; ****, *P* < 0.0001; ns, not significant), while the comparisons between the inhibitory activities of IPB02V3 and IPB24 were by *t*-test, in which *P *< 0.05 is considered as a significant difference.

## Results

### Functional characterization of S proteins of divergent SARS-CoV-2 variants

**Figure 2.**
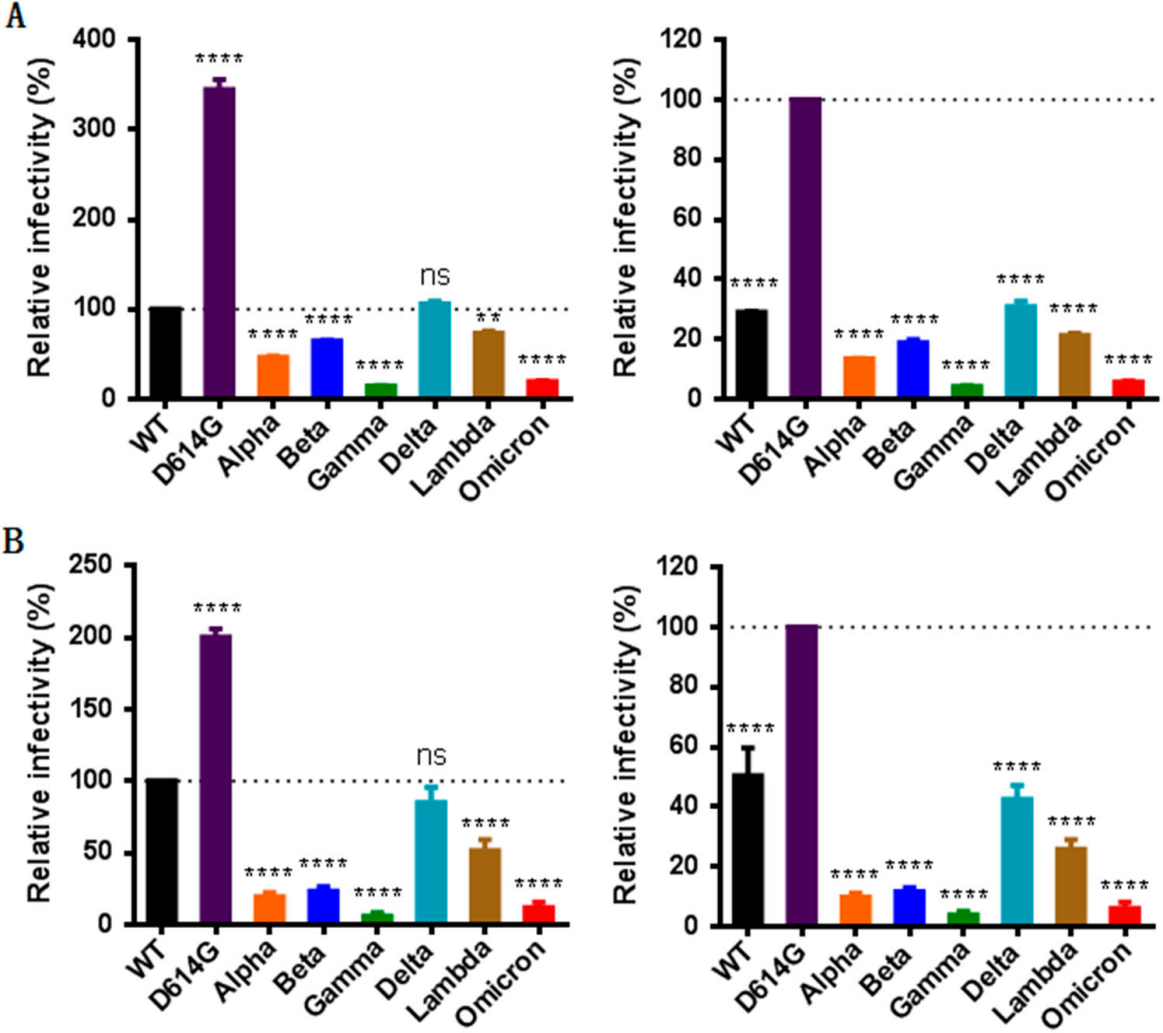
Infectivity of divergent SARS-CoV-2 variants determined by a single-cycle infection assay. The infectivity of S protein-pseudotyped SARS-CoV-2 variants was determined in 293T/ACE2 cells (A) and Huh-7 cells (B). In comparison, the luciferase activity (RLU) of wild-type (WT) (left panels) or D614G mutant (right panels) was treated as 100% and the relative infectivity of other pseudoviruses were calculated accordingly. Data were derived from the results of three independent experiments and are expressed as means with standard deviations (SD).

We first determined the impact of mutations on the functionality of S protein to mediate virus’s infectivity. To this end, a panel of pseudoviruses carrying the S proteins of divergent VOCs (Alpha, Beta, Gamma, Delta, and Omicron) and a variant of interest (VOI, Lambda) were generated and their infectivity on 293T/ACE2 and Huh-7 cells were measured by a single-cycle infection assay. Consistent with our previous findings [12], the single D614G mutant exhibited markedly increased infectivity relative to wild-type (WT) strain; however, various variants except Delta had significantly decreased infectivity on both target cells (Figure 2, left panels). Given that all the current variants evolved with the D614G mutation, we also analysed the functions of divergent S proteins with the D614G mutant as a reference strain. As shown in the right panels of Figure 2, all the variants including the Delta strain displayed dramatically reduced cell entry capacities as compared to the D614G pseudovirus infection. These results suggested that the S proteins of SARS-CoV-2 variants possess a reduced ability to mediate infection when packaged in the pseudotyped virions, and especially, the S proteins of the Omicron and Gamma strains show the lowest infectivity.

**Figure 3.**
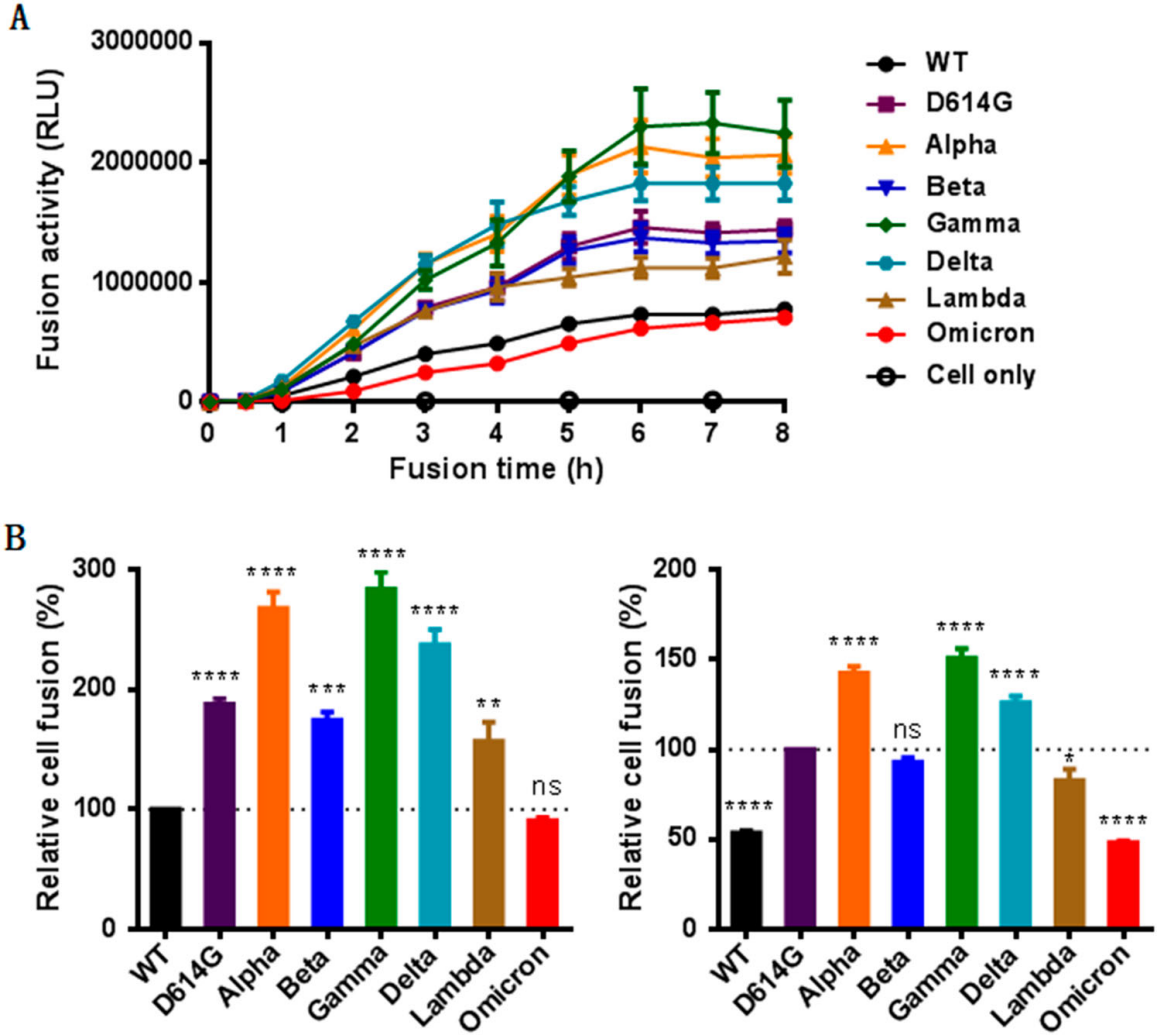
The fusion activity of divergent SARS-CoV-2 variants determined by a DSP-based cell fusion assay. (A) Fusion kinetics of divergent S proteins in 293T/ACE2 cells was determined at different time points. (B) The relative fusion activities of divergent S proteins at 8 h postfusion were calculated with WT (left panel) or D614G mutant (right panel) as a reference.

We next sought to characterize the S proteins to mediate cell–cell membrane fusion by a DSP-based cell fusion assay. As shown in Figure 3(A), the S protein-expressing 293T effector cells rapidly fused with 293T/ACE2 target cells, approaching a plateau after mixing the cells 6 h. We then compared the cell fusion activities of the S proteins at 8 h postfusion. Interestingly, all the variants except Omicron exhibited markedly enhanced fusion capacities relative to WT (Figure 3(B), left panel). When D614G was used as a reference, the Alpha, Gamma, and Delta variants remained the increased fusion, the Beta variant did not show the difference, whereas the Omicron and Lambda variants had significantly decreased fusion activities (Figure 3(B), right panel). Therefore, only the S proteins of D614G and Omicron displayed accordant fusion results with the infection of the above pseudoviruses, the mechanism underlying the discrepancy with other variants (Alpha, Beta, Gamma, Delta, and Lambda) requires further characterizations.

### IPB02V3 and IPB24 maintain high potency against Omicron and other variants

**Figure 4.**
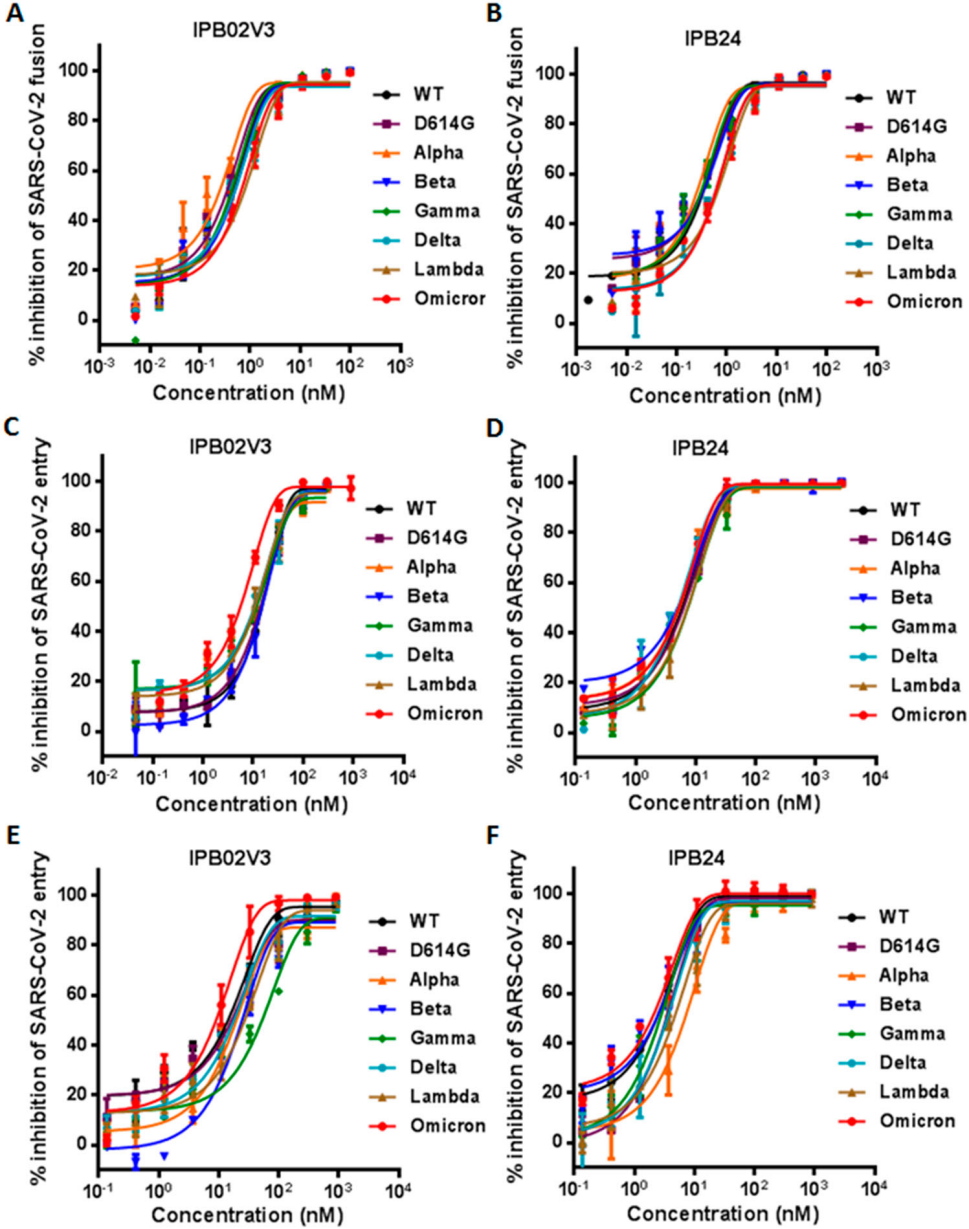
Inhibitory activity of IPB02V3 and IPB24 against SARS-CoV-2 variants. The inhibitory activities of IPB02V3 (A) and IPB24 (B) on the cell–cell fusion mediated by S proteins of divergent SARS-CoV-2 VOCs (Alpha, Beta, Gamma, Delta, and Omicron) and VOI (Lambda) were determined by a DSP-based cell fusion assay. The inhibitory activities of IPB02V3 and IPB24 against the infections of divergent VOCs and VOI pseudoviruses in 293T/ACE2 cells (C and D) or Huh-7 cells (E and F) were determined by a single-cycle infection assay. Samples were tested in triplicate, the experiments were repeated three times, and data are expressed as the means with SD.

We next focused on assessing the activities of two representative fusion inhibitor lipopeptides, IPB02V3 and IPB24, in inhibiting Omicron and other variants. First, their inhibitory activities against S protein-mediated cell–cell fusion were measured by the dual-DSP-based cell fusion assay. As shown in Figure 4(A,B) and Table 1, both IPB02V3 and IPB24 inhibited the cell fusion mediated by various S proteins with comparable activities, and specifically, they potently blocked the Omicron S protein in a dose-dependent manner with IC_50_ values of 0.61 and 0.51 nM, respectively. We subsequently determined the inhibitory potencies of IPB02V3 and IPB24 against divergent pseudoviruses by the single-cycle infection assay. As shown in Figure 4(C–F) and Table 1, two lipopeptides maintained the very potent activities in inhibiting the viruses, and interestingly, IPB02V3 exhibited an improved activity against Omicron infection on 293T/ACE2 but not Huh-7 cells. Specifically, IPB02V3 inhibited the WT and Omicron pseudoviruses on 293T/ACE2 cells with the IC_50_s of 18.53 and 6.35 nM, respectively, and inhibited the two viruses on Huh-7 cells with the IC_50_s of 16.29 and 11.52 nM, respectively. In comparison, IPB24 was a more active inhibitor of VOCs or VOI strains than IPB02V3 in inhibiting pseudovirus infection. These results demonstrated that both IPB02V3 and IPB24 lipopeptides remain the highly potent inhibitors of Omicron and other variants, and that IPB02V3 even has increased potency against Omicron relative to the WT and various VOCs.

**Table 1.** Inhibititory activity of lipopeptide fusion inhibitors against divergent SARS-CoV-2 variants (IC_50 _± SD)^a^.

SARS-CoV-2	Cell fusion			Pseudovirus on 293T/ACE2			Pseudovirus on Huh-7		
	IPB02V3	IPB24	*P* value	IPB02V3	IPB24	*P* value	IPB02V3	IPB24	*P* value
WT	0.38 ± 0.01	0.36 ± 0.02	>0.05	18.53 ± 1.70	5.51 ± 0.54	<0.01	16.29 ± 1.26	2.43 ± 0.07	<0.001
D614G	0.35 ± 0.03	0.33 ± 0.03	>0.05	17.00 ± 2.15	6.17 ± 0.05	<0.05	18.57 ± 1.61	3.15 ± 0.06	<0.001
Alpha	0.24 ± 0.02	0.22 ± 0.001	>0.05	19.10 ± 3.69	5.94 ± 0.19	<0.05	14.03 ± 1.81	5.42 ± 0.30	<0.01
Beta	0.45 ± 0.02	0.36 ± 0.01	<0.05	16.64 ± 0.04	5.65 ± 0.01	<0.001	18.02 ± 3.88	3.29 ± 0.76	<0.01
Gamma	0.34 ± 0.04	0.27 ± 0.004	>0.05	17.54 ± 1.75	6.41 ± 0.17	<0.05	24.52 ± 7.02	3.95 ± 0.71	<0.01
Delta	0.42 ± 0.03	0.48 ± 0.02	>0.05	12.81 ± 1.63	4.94 ± 0.05	<0.05	15.37 ± 3.73	3.46 ± 0.13	<0.01
Lambda	0.78 ± 0.01	0.68 ± 0.04	>0.05	16.72 ± 0.70	6.57 ± 0.02	<0.01	19.50 ± 7.29	4.10 ± 0.69	<0.05
Omicron	0.61 ± 0.07	0.51 ± 0.08	>0.05	6.35 ± 0.75	4.51 ± 0.30	>0.05	11.52 ± 3.20	2.56 ± 0.47	<0.01

^a^ The experiments were performed in triplicate and repeated three times, and data are expressed as the means ± SD.

### IPB02V3 and IPB24 are more effective inhibitors of authentic Omicron infection

**Figure 5.**
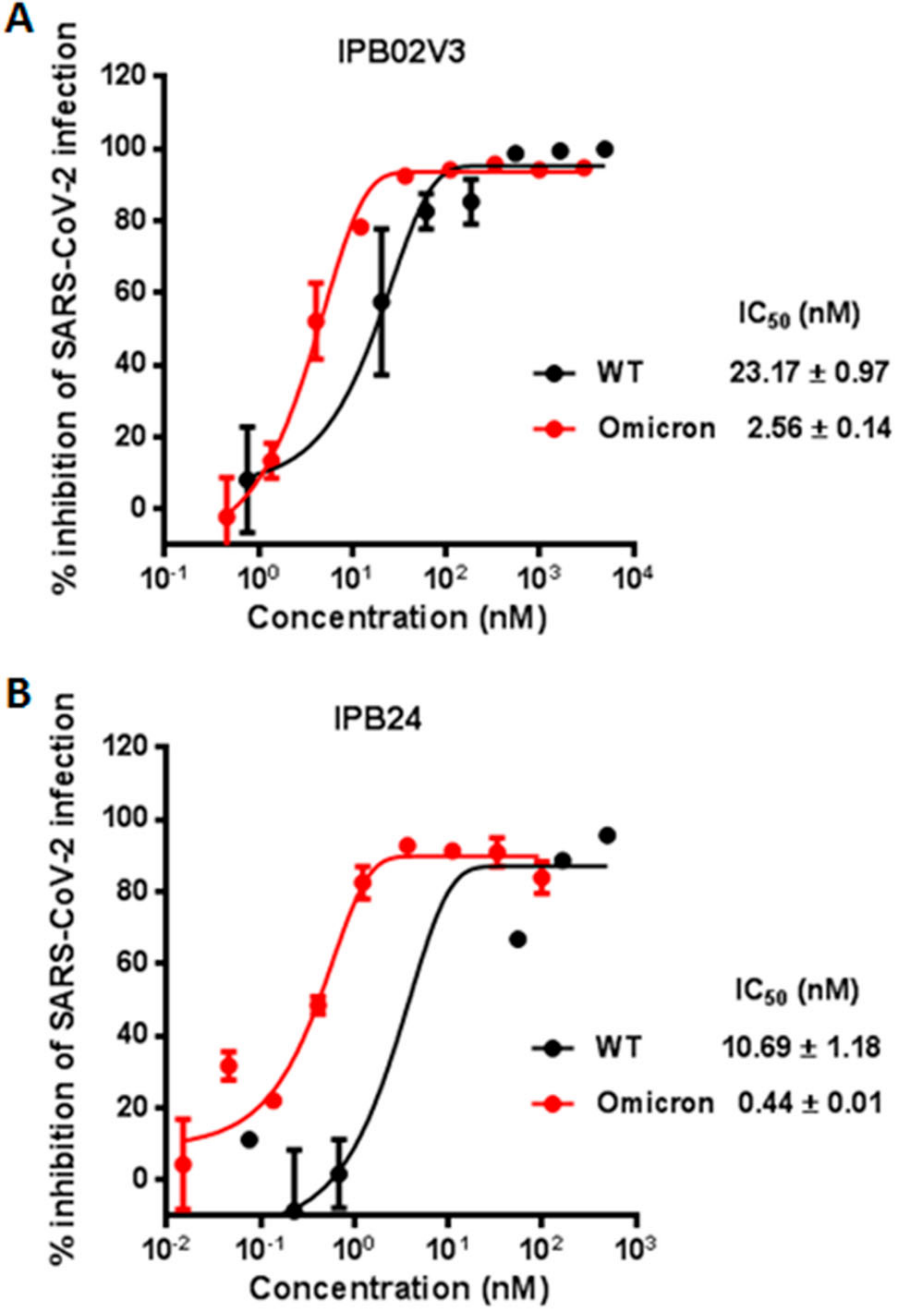
Inhibitory activities of IPB02V3 and IPB24 against live SARS-CoV-2 infection. The inhibitory activities of IPB02V3 (A) and IPB24 (B) against the infections of authentic SARS-CoV-2 WT and Omicron strains were determined in Vero cells. The experiments were repeated at least two times, and data are expressed as the means ± SD.

Given that IPB02V3 and IPB24 could effectively inhibit the Omicron S-mediated cell–cell fusion and pseudovirus infection, we were intrigued to determine their inhibitory activities on the infection of live Omicron variant. To this end, a live WT strain was also tested for comparison. Surprisingly, it was found that IPB02V3 inhibited WT and Omicron with IC_50_s of 23.17 and 2.56 nM, respectively, which indicated a 9.05-fold increase for the susceptibility of Omicron relative to WT (Figure 5(A)); IPB24 inhibited WT and Omicron with IC_50_s of 10.69 and 0.44 nM, respectively, which indicated a 24.3-fold increased susceptibility for Omicron over the WT strain (Figure 5(B)). Thus, both IPB02V3 and IPB24 lipopeptides are more potent inhibitors of authentic Omicron infection as compared to the inhibition on the original SRAS-CoV-2 (Wuhan-Hu-1 strain).

## Discussion

In summary, we first characterized the functionalities of the S proteins possessing by various SARS-CoV-2 variants, and found that S protein-pseudotyped viruses had greatly decreased infectivity relative to the original wild-type (WT) strain and a single D614G mutant, especially did by the Omicron pseudovirus. However, while the S protein of Omicron still exhibited relatively reduced activity on cell–cell fusion, all other variants evolved with the S protein behaving a significantly enhanced fusogenic function. We then determined the susceptibility of divergent S proteins to the inhibition of two representative fusion inhibitors by the DSP-based cell–cell fusion assay and pseudovirus-based single-cycle infection assay. The results verified that both IPB02V3 and IPB24 lipopeptides maintained the highly potent activities in inhibiting diverse S proteins-driven cell–cell fusion and pseudovirus infection. Furthermore, we surprisingly found that live Omicron virus was more sensitive than the WT strain to the inhibition by IPB02V3 or IPB24. Thus, we conclude that SARS-CoV-2 HR2-derived lipopeptides are very potent and broad-spectrum fusion inhibitors against Omicron and other divergent variants.

As of 19 May 2022, SARS-CoV-2 has globally led to about 521 million confirmed cases of COVID-19 and caused over 6.27 million deaths, thus severely impacting the global public health and socioeconomic stability (https://covid19.who.int). Although more than 12 billion vaccine doses have been administrated worldwide, the emergence of SARS-CoV-2 variants, including divergent VOC and VOI strains, poses new challenges to the disease countermeasures, such as vaccine prevention and therapeutic strategies [1,2,15–18]. The Omicron variant was first reported from Southern Africa at the late 2021 but it has now dominated the pandemic, calling for the urgent development of vaccines and drugs that have a broad-spectrum antiviral activity [5,6,19–21]. The Omicron variant evolves with more than 50 mutations, by far the largest number of mutations among all SARS-CoV-2 variants. Importantly, there are 32 mutations located within S protein, which almost cover all the key mutations of the previous VOCs, including the K417N, E484A, and N501Y mutations in the receptor-binding domain (RBD) of S1 subunit and other known mutations that have been proved to change the sensitivity of the virus to neutralizing antibodies [4,5,15,22]. Since the emergence of the D614G mutant, the impacts of S protein mutations on the infectivity and antigenicity of SARS-CoV-2 have been specifically focused [2,17,23]. Different from the Delta and other variants that showed enhanced fusogenicity and infectivity [24,25], the Omicron variant exhibited decreased cell fusion activity in different experimental systems and conditions [5,20,26]. Herein, our studies verified the reduced fusogenicity and infectivity of Omicron, but the mechanism underlying the inconsistence between the fusogenicity and infectivity from other VOCs remains elusive and needs characterization in details. Moreover, how the Omicron variant evolves with reduced fusogenicity but behaves with dramatically increased transmissibility is also an intriguing question to be elucidated.

In the past decade, we have dedicated to the development of lipopeptide-based viral fusion inhibitors [27–35]. Different from those unconjugated native HR2 peptides, the lipopeptide inhibitors can enable activity against virus entry from both the cell surface and endosomal pathways [36,37]. As reported, we initially designed a potent SARS-CoV-2 fusion-inhibitory lipopeptide termed IPB02 [11]. The antiviral activity of IPB02 could be greatly improved by sequence optimization and introduction of a flexible linker between the peptide sequence and lipid group, as evidenced by IPB02V3 [12]. Later, we found that the S protein membrane-proximal external region (MPER) critically determines the SARS-coV-2 infectivity and accordingly designed a panel of lipopeptides (IPB20∼IPB27) containing the MPER sequence [13]. As shown by IPB24, the newly developed inhibitors possessed highly potent activities in inhibiting SARS-CoV-2 bearing S protein with the mutations of D614G, E484K, N501Y, Δ69–70, N501Y/Δ69–70/P681H, or N501Y/E484K/K417N [13]. Compared to the S1 subunit, the fusogenic S2 subunit of SARS-CoV-2 is more conserved during the virus evolution; however, three mutations (Q954H, N969K, and L981F) also exist in the HR1 site of Omicron. It was reported that none of these HR1 mutations affected the interaction between the fusion inhibitor EK1 and an HR1-derived target mimic peptide and the antiviral activity of EK1-based lipopeptides [38,39], which were consistent with our results presented here. Given that IPB02V3 and IPB24 target the different sites and possess a more active inhibitory activity than the EK1-based inhibitors [12,13], they offer ideal candidates for the development of clinically applicable antivirals against the Omicron variant. Moreover, it is conceivable that IPB02V3 and IPB24 could be effective countermeasures in fighting against a potential future outbreak of new SARS-CoV-2 variants that emerge during the ongoing COVID-19 pandemic worldwide.

## Data Availability

All data are fully available without restriction.
